# Dietary Quality Indices in Early Pregnancy and Rate of Gestational Weight Gain among a Prospective Multi-Racial and Ethnic Cohort

**DOI:** 10.3390/nu15040835

**Published:** 2023-02-06

**Authors:** Emily F. Liu, Yeyi Zhu, Assiamira Ferrara, Monique M. Hedderson

**Affiliations:** Division of Research, Kaiser Permanente Northern California, Oakland, CA 94612, USA

**Keywords:** gestational weight gain, pregnancy, mothers, diet quality, dietary pattern

## Abstract

Meeting the Institute of Medicine (IOM) gestational weight gain (GWG) guidelines is associated with a reduced risk of adverse perinatal outcomes. Overall diet quality comprehensively assesses dietary components and accounts for interactions between them. While GWG is influenced by maternal diet, its association with overall diet quality—measured by various dietary quality indices—is not well-defined. We prospectively estimated the relationship between four established dietary quality indices and the risk of GWG rate above (excessive) or below (inadequate) IOM guidelines in a multi-racial and ethnic cohort of 2914 pregnant people from the Pregnancy Environment and Lifestyle Study (2014–2019). We assessed diet quality using the Healthy Eating Index 2010 (HEI-2010), alternate Mediterranean Diet (aMED), Dietary Approaches to Stop Hypertension (DASH), and Empirical Dietary Inflammatory Index (EDIP). Following the first trimester, 56% of the cohort had excessive GWG, and 14% had inadequate GWG. Poor diet quality (below the 75th percentile), measured by HEI-2010, was associated with a higher risk of excessive GWG in the second and third trimesters [RR = 1.03 (1.00, 1.06)]. Effect modification of this relationship by race and ethnicity and pre-pregnancy BMI was assessed. We found poor diet quality to be associated with elevated risk of excessive GWG among Black participants [RR = 1.14 (1.02, 1.28)] and White participants [RR 1.07 (1.01, 1.12)]. This was also the case for participants with pre-pregnancy BMI < 25.0 [RR 1.05 (1.00, 1.10)]. These results suggest that diet quality measured by the HEI-2010 is associated with excessive GWG, and the associations appear to be stronger among pregnant people without overweight or obesity and pregnant people who identify as Black or White race and ethnicity.

## 1. Introduction

Excessive or inadequate weight gain during pregnancy is associated with adverse maternal and perinatal outcomes. In order to minimize risk and promote mother and child health, the Institute of Medicine (IOM)—now known as the National Academy of Medicine—set guidelines for appropriate gestational weight gain (GWG) [1]. Excessive GWG is associated with obstetric and maternal health outcomes such as cesarean deliveries and post-partum weight retention [2] and subsequent development of overweight and obesity [3]. Infants of excessive GWG pregnancies are more likely to be born large for gestational age and to have overweight or obesity later in childhood [4,5,6]. Infants of inadequate GWG pregnancies are more likely to be born small for gestational age and to have low birth weight [2].

Between 2011 and 2012, 48 percent of pregnant people in the United States exceeded the recommended range for GWG, and only one-third met recommendations [7]. Individuals with pre-pregnancy obesity are at a higher risk of excessive GWG compared to their counterparts with lower BMI [8]. Given the increasing prevalence of obesity in the United States and many other parts of the world, the population at risk for excessive GWG is expected to grow.

Diet is a potentially modifiable risk factor for helping to achieve optimal GWG and can be assessed using dietary patterns. Previous studies on diet during pregnancy have focused largely on isolated foods or nutrients in relation to GWG [9]. However, dietary patterns may better account for the likely synergism and interaction between foods and nutrients. Many dietary guidelines now focus on healthy eating patterns, rather than on individual nutrients. Higher-quality diets measured using established dietary quality indices, such as the Healthy Eating Index (HEI), Dietary Approaches to Stop Hypertension (DASH), and alternate Mediterranean Diet (aMED), have been associated with less long-term weight gain compared to their lower-scoring counterparts among non-pregnant individuals [10]. In comparison, the relationship between these dietary quality indices and GWG is less well-defined. The United States Department of Agriculture and the Department of Health and Human Services created a joint initiative called “Pregnancy and Birth to 24 Months” to conduct a series of systematic reviews on diet and health among pregnant people, and the review concluded that that evidence was limited for dietary patterns and maternal health outcomes [11]. Few studies have been conducted, and no association has been found between the HEI and GWG. These studies have been limited to cross-sectional data or small sample sizes with minimal racial and ethnic diversity, and to our knowledge, none assess dietary quality indices beyond the HEI [12,13]. Additionally, novel dietary patterns such as the Empirical Dietary Inflammatory Pattern (EDIP) [14], an index of a pro-inflammatory diet, have been associated with weight change in adult populations but have not been assessed in relation to GWG [15].

More conclusive evidence for the HEI and other dietary quality indices is needed to support people in meeting their weight gain goals during pregnancy. The primary aim of this study was to prospectively assess the relationship between diet quality in the first trimester and subsequent GWG rate in the second and third trimesters and to evaluate whether this relationship was modified by pre-pregnancy body mass index (BMI) and race and ethnicity for multiple dietary quality indices (HEI, DASH, aMED, and EDIP).

## 2. Materials and Methods

### 2.1. Study Population

The study population was derived from the Pregnancy Environment and Lifestyle Study (PETALS). PETALS is a longitudinal multi-ethnic birth cohort of mother-infant pairs, and its study design and scope have been reported elsewhere [16]. Between April 2014 and May 2019, study participants were recruited before gestational week 11 at five medical centers within Kaiser Permanente Northern California. Patients were eligible if they were carrying singleton infants, 18–45 years of age, and did not have recognized pre-existing diseases of diabetes, cancer, hepatitis C, or liver cirrhosis. The participation rate for PETALS was 75 percent [16]. Survey data were collected at study clinic visit 1 (gestational weeks 10–13) and clinic visit 2 (gestational weeks 16–19). The study was approved by the human subjects committee of the Kaiser Foundation Research Institute. Informed consent was obtained from all participants.

**Figure 1:**
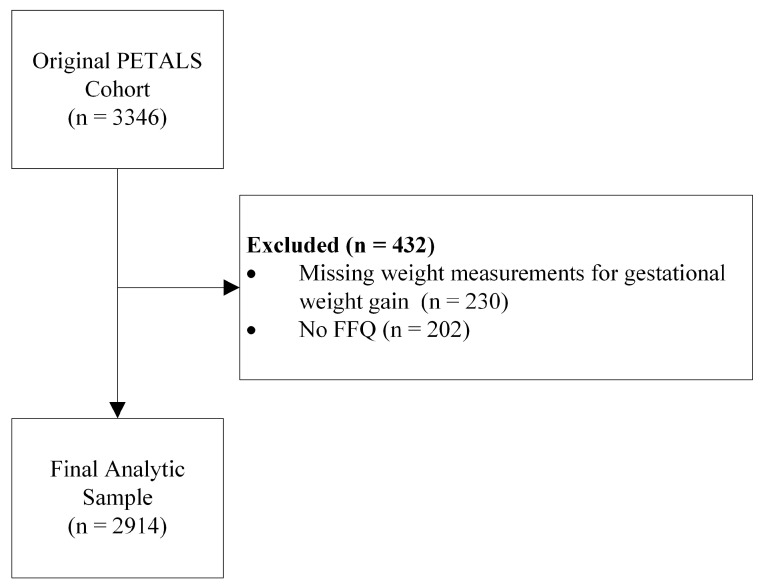
Flow diagram for primary analysis cohort.

A total of 3346 eligible patients were enrolled in the PETALS study at the time of analysis. There were a total of 2914 participants who completed a food frequency questionnaire (FFQ) at study clinic visit 1 and had weight data at three time points: before pregnancy, at study clinic visit 1, and delivery (Figure 1).

### 2.2. Study Variables

#### 2.2.1. Diet Quality Indices

Dietary intake was assessed using the Block FFQ at study clinic visit 1 [17]. Participants were asked to recall the average portion size and consumption frequency of foods and beverages over the last three months. The Block FFQ has been validated using multiple diet records, and since its development, has been used with diverse populations, including pregnant people [18]. Nutrient intakes were adjusted for total energy intake using the residual method [19]. We used the Block FFQ to calculate diet quality scores for four dietary quality indices: Healthy Eating Index 2010 (HEI-2010), Dietary Approaches to Stop Hypertension (DASH), alternate Mediterranean Diet (aMED), and Empirical Dietary Inflammatory Pattern (EDIP). We excluded alcohol intake in diet quality scores because its consumption is not recommended for pregnant people and therefore is unlikely to be accurately reported [20].

Our study period was equally covered by the HEI-2010 and HEI-2015 guidelines and we chose to use the HEI-2010 for this study to align with prior studies using this cohort [21]. The HEI-2010 dietary quality index comprises 12 components, each worth 5 to 20 points, with a total maximum score of 100. There are nine adequacy components (i.e., recommended increased intake): total fruit, whole fruit, total vegetables, greens and beans, whole grains, dairy, total protein foods, seafood and plant proteins, fatty acids; and three moderation components (i.e., recommended decreased intake): refined grains, sodium, and empty calories [22]. We excluded alcohol from the empty calories component. DASH is a dietary index that focuses on foods and nutrients associated with the prevention and control of hypertension. There are 8 components, each worth 1 to 5 points, with a total maximum score of 40. Adequacy components are fruits, vegetables, nuts and legumes, low-fat dairy products, and whole grains; moderation components are sodium, red and processed meats, and sweetened beverages [23]. The aMED dietary index is an adaptation of the Mediterranean diet to non-Mediterranean countries. There are typically 9 components each worth 0 or 1 point with a total maximum score of 9. Components include alcohol, red and processed meat, fish, whole grains, legumes, nuts, fruits, vegetables, and monounsaturated to saturated fat ratios. However, our cohort had a total maximum score of 8 because we excluded the alcohol component [24]. A higher score for the HEI-2010, DASH, and aMED indicates better alignment with recommendations and higher diet quality.

We also examined the EDIP which is an empirically driven pattern that focuses on the inflammatory potential of overall diet [14]. This is different from the HEI-2010, DASH, and aMED, which are a priori indices and assess the quality of overall diet. The EDIP has 18 components and is calculated by summing the weighted mean daily intake for each component. Nine components are considered inflammatory, including processed meat, red meat, organ meat, other fish, other vegetables, refined grains, high-energy beverages, low-energy beverages, and tomatoes; and nine are considered anti-inflammatory, including beer, wine, tea, coffee, dark yellow vegetables, leafy green vegetables, snacks, fruit juice, and pizza. Again, we excluded alcohol components due to recommendations for pregnant people. There is no maximum score for EDIP; smaller values indicate a less inflammatory diet, while larger values indicate diets with more inflammatory potential.

Diet quality was dichotomized into “good” and “poor” diet quality. Dietary quality indices where higher values indicated better alignment with recommendations (i.e., HEI-2010, DASH, and aMED) defined good diet quality as the highest quartile. In contrast, we used the lowest quartile to denote good diet quality for the EDIP, where lower values indicate less inflammation.

#### 2.2.2. Rate of Gestational Weight Gain

Gestational weight gain was defined as the difference between weight measurements at delivery and study clinic visit 1. Rate of GWG was calculated by dividing GWG by the number of weeks between the two measurements. Values were then categorized into inadequate, adequate, or excessive based on the IOM-recommended ranges that varied depending on pre-pregnancy body mass index (BMI).

#### 2.2.3. Covariates

Demographic and medical information was obtained through structured questionnaires during study clinic visit 1. Covariates included maternal age (18–24, 25–29, 30–34, ≥35 years); education level (≤high school, high school/GED, some college, ≥college); household income (USD < 50,000, USD 50,000–99,999, USD 100,000–149,999, USD ≥ 150,000); nulliparity, physical activity (metabolic equivalent of task (METs) per week); total energy intake during pregnancy (kcal per day) and additional dietary intake data from the FFQ. Race and ethnicity were categorized into five groups: non-Hispanic White (White), non-Hispanic Black (Black), Asian and Pacific Islander, Hispanic, and other. People who identified as multi-racial or had an unknown race and ethnicity were placed into the other race and ethnicity category due to the small sample size.

Weight measurements were extracted from the electronic health record (EHR). Pre-pregnancy BMI was calculated as pre-pregnancy body weight (kilograms) divided by height (meters) squared. Pre-pregnancy body weight was defined as a measured weight within 12 months before pregnancy (78%). If a measurement was not available in this timeframe, a self-reported pre-pregnancy weight or pregnancy weight measured before 10 weeks’ gestation was used (22%). Pre-pregnancy BMI was categorized into three groups to examine effect modification (BMI < 25.0, 25.0–29.9, ≥30.0).

### 2.3. Statistical Analysis

All study variables were summarized using descriptive statistics. Continuous variables are presented as mean and standard deviation; categorical variables are presented as frequencies.

Multivariable Poisson regression models with robust standard errors estimated the relative risk (RR) and 95% confidence intervals (CIs) separately comparing (1) excessive rate of GWG to adequate rate of GWG and (2) inadequate rate of GWG to adequate rate of GWG in relation to each dietary quality index dichotomized into good and poor diet quality. Models were adjusted for physical activity, education level, parity, household income, race-ethnicity, age at delivery, pre-pregnancy BMI, and total energy intake. Effect modification by pre-pregnancy BMI and race and ethnicity were, respectively, assessed using an interaction term and a P for interaction. Stratified models were examined among diet indices with a P for interaction less than 0.2. Separate models were used for each dietary quality index. Models were adjusted for the same covariates with the exception of the stratifying variable. Sensitivity analysis examined dietary index scores as quartiles for both excessive and inadequate rate of GWG.

All analysis was conducted in SAS Studio v3.81.

## 3. Results

Table 1 presents the characteristics of the study population. Among 2914 pregnancies in the PETALS cohort, 30% met gestational weight gain recommendations. The population was racially and ethnically diverse: 24% Asian and Pacific Islander, 9.6% Black, 40% Hispanic, 3.2% of other racial/ethnic groups, and 23% were non-Hispanic White. On average, pre-pregnancy BMI was 27.0 (±6.0) kg/m^2^. Of the cohort, 46% were nulliparous, and 36.8% were between 30 and 34 years old. The majority had at least a high school degree, and there was a range of household incomes represented in the population. The average diet index score was 71 (±10) for HEI-2010, 24 (±4.4) for DASH, 4 (±1.79) for aMED, and −0.05 (±0.45) for EDIP.

**Figure 2:**
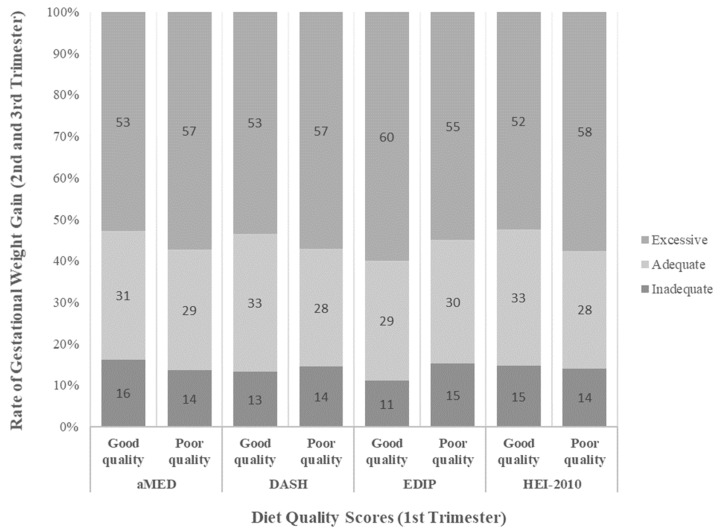
Proportion of inadequate, adequate, and excessive rate of gestational weight gain by diet quality defined by aMED, DASH, EDIP, and HEI-2010.

The proportion of rate of GWG significantly differed by good and poor diet quality for each dietary quality index (Chi^2^ *p* < 0.05). With the exception of EDIP, good-quality diets—compared to poor-quality diets—in the first trimester had a slightly higher proportion of participants with adequate GWG rate and a smaller proportion with excessive GWG rate in the second and third trimesters (Figure 2).

The relative risks of excessive and inadequate GWG by diet quality are presented in Table 2. Overall, poor diet quality assessed by the HEI-2010 was associated with higher risk of excessive GWG. Participants with poor diet quality had a 3% [95% CI 1.00, 1.06] increased risk of excessive GWG rate in their second and third trimesters. No significant associations were observed for dietary quality indices of aMED, DASH, and EDIP in relation to risk of excessive GWG rate. When examining the relative risk of inadequate GWG by diet quality, there were no significant associations found among all four dietary quality indices. When examining full model results, pre-pregnancy BMI was the only covariate significantly associated with excessive GWG and inadequate GWG across all four dietary quality indices.

Sensitivity analyses examining diet quality indices as quartiles supported results from the primary analysis and found the lowest two quartiles of diet quality for HEI-2010 to have increased risk of excessive GWG compared to the highest quartile [RR = 1.04 (1.00, 1.07) and RR = 1.05 (1.01, 1.08), respectively]. There were no other significant associations (Supplementary Tables S1 and S2).

There was suggestive effect modification by pre-pregnancy BMI for the HEI-2010 (*p* for interaction ≤0.001). Forty-four percent of the cohort had a pre-pregnancy BMI less than 25.0. The majority had overweight (25.0–29.9) or obese (≥30.0) pre-pregnancy BMI (data not shown). In stratified analyses, participants with normal pre-pregnancy BMI and poor diet quality had a 5% [95% CI 1.00, 1.09] higher risk of excessive GWG. There were no significant associations found among participants with an overweight or obese pre-pregnancy BMI (Table 3).

There was also evidence of effect modification by race and ethnicity for the HEI-2010 (P for interaction = 0.02). Descriptively, White participants had the lowest proportion of adequate GWG (28%) compared to all other racial and ethnic groups. In stratified analyses, poor diet quality was generally associated with higher risk of excessive GWG among Black participants [RR = 1.14 (1.02, 1.28)] and White participants [RR 1.07 (1.01, 1.12)]. There was no association between HEI-2010 and excessive GWG among Asian and Pacific Islander, Hispanic, or other racial and ethnic group participants (Table 4).

## 4. Discussion

This multi-ethnic prospective cohort study found that poorer diet quality in early pregnancy, measured by the HEI-2010, was associated with elevated risk of exceeding IOM guidelines for GWG rate, with stronger associations, respectively, observed among pregnant people without overweight or obesity and pregnant people who identify as Black or White race and ethnicity. There was no association between the DASH, aMED, and EDIP and excessive GWG. In addition, no dietary pattern was associated with inadequate GWG.

Past studies examining the associations between diet quality assessed via HEI and gestational weight gain have been conflicting. Several studies that were limited to cross-sectional data or small sample sizes [12,13,25,26,27] found no link between diet quality and GWG. However, most of these studies did not examine the prospective association between diet quality and subsequent GWG. The study by Schlaff et al. [13] was the only one that prospectively examined diet quality in the second and third trimesters in relation to subsequent GWG. While no association was observed, it could be due to the small sample size (41 participants) or the shorter time interval between diet assessment and GWG [13]. In our primary analysis, the majority of our findings were null, and those that were significant were small in magnitude. Assuming that our observed association between HEI-2010 and excessive GWG is a true effect, it is possible that prior studies were insufficiently powered to detect an overall effect and even less so for subgroups.

Our findings are consistent with other prospective studies with larger sample sizes that have examined dietary patterns similar to the HEI. Parker et al. [28] examined differences in mean Alternative Healthy Eating Index for Pregnancy scores among pregnant people who had inadequate, adequate, and excessive GWG among a sample of 908 people and found lower diet quality score to be associated with excessive GWG. Similarly, in a prospective cohort of 1113 pregnant people in Sweden, Augustin et al. [29] found that those with a poor or fair diet quality, on average, gained 2 kg more than those with higher-quality diets following the Swedish National Food Agency recommendations.

Pregnant people with overweight or obesity are more likely to exceed GWG according to the IOM guidelines [30]. We examined effect modification by pre-pregnancy BMI and found that diet quality was associated with excessive GWG only among participants with a pre-pregnancy BMI < 25.0 and not participants with overweight or obesity. This finding is consistent with broader scientific literature that has found health behavior and lifestyle interventions focused on diet and physical activity for GWG to be less effective among pregnant people with overweight and obesity [7]. It is hypothesized that the decrease in insulin sensitivity during pregnancy, compounded by pre-existing metabolic conditions among people with overweight and obesity (i.e., decreased insulin sensitivity and increased adiposity), may make them less responsive to changes initiated during the relatively short pregnancy period [31].

We also found that the association between diet quality and excessive GWG varied across racial and ethnic groups. Prior evidence supports differences in excessive GWG by racial and ethnic group [32,33], with Black and White individuals having the highest prevalence of excess GWG. However, the reasons underlying these racial and ethnic differences are not well understood. Our results showed a stronger relationship between diet and subsequent excessive GWG among Black and White participants and no significant associations among Hispanic, Asian and Pacific Islander, and other racial and ethnic group participants. Compared to the overall analysis, the effect size was considerably greater for Black participants in the race-stratified models. The effect size for White participants was modest in comparison. Past studies have suggested that there may be racial and ethnic differences in micronutrient and macronutrient intake [34,35,36], and we speculate that our finding could be partly due to variation in energy and nutrient sources and their impact on GWG. It is also possible that there are unmeasured contextual factors, such as environmental or economic conditions, that covary with different racial and ethnic groups.

Mechanisms of diet quality and GWG have yet to be explained. Prior studies have found higher diet quality measured by HEI to be associated with greater fiber and lower refined carbohydrate intake [37]. This dietary intake profile may increase satiety and reduce overeating [38]. It could also lower glycemic response [39]. These changes could be linked to an improved metabolic profile and, in turn, influence GWG. While this hypothesis is plausible, the small overall effect size in our study suggests that there are also other factors driving GWG. When examining effect modification by race and ethnicity, the larger effect size among Black and White participants suggests that the relationship of interest may also be influenced by a variety of individual and contextual factors that need to be further disentangled. A better understanding of how diet quality differentially impacts GWG could help explain persistent racial and ethnic disparities in maternal and fetal health.

We urgently need to understand how to support pregnant people in meeting the IOM GWG guidelines as the majority of pregnancies exceed the current GWG guidelines in the United States. From a clinical perspective, results of this study suggest that good diet quality measured by HEI-2010 was the only dietary quality index associated with excessive GWG, and therefore, it may be the best available dietary pattern to recommend during pregnancy. However, given the strength of the association, following the USDA Dietary Guidelines for Americans is not sufficient to fully address excessive or inadequate GWG. Health care professionals should consider diet quality to be one of many factors when providing recommendations for pregnant patients. We also found that increasing pre-pregnancy BMI was a risk factor of excessive GWG across all four dietary indices. If health care professionals have the opportunity to work with patients during the preconception period, lifestyle interventions to support patients in reaching a healthy BMI before pregnancy could play a substantial role in improving GWG.

Our study is one of few that have found an association between diet quality and gestational weight gain. We had several strengths in our analysis, including our large sample size and prospective data on dietary quality in early pregnancy and subsequent rate of GWG according to the IOM guidelines for rate of GWG. Our sample size allowed us to adequately explore effect modification by pre-pregnancy BMI and race and ethnicity through stratified models. Yet, despite these strengths, our study had several potential limitations. First, we relied on self-reported FFQ data to assess dietary intake in the first trimester of pregnancy. While the FFQ has been validated against four-day diet records, it may still be vulnerable to recall bias and misclassification [18]. Second, we focused on diet quality in the first trimester and did not have dietary data for mid-to-late pregnancy, which may play a larger role in fetal growth and that may impact total gestational weight gain. However, there is consistent evidence that maternal diet pattern does not change significantly over the course of pregnancy [40,41]. Furthermore, our cohort had a mean score of 71 for the HEI-2010, exceeding the total score for the US population of 59 [22,42]. Therefore, our cohort may have healthier dietary habits compared to the general population of pregnant people in the US.

Diet represents a potential opportunity to intervene during pregnancy, and future studies should explore diet quality by conducting further analyses within racial and ethnic groups and pre-pregnancy BMI groups to understand the mechanisms between diet quality and GWG. We also recommend examination of modifiable factors beyond diet quality and physical activity to identify drivers of GWG to support healthy pregnancy weight gain. Finally, given the influence of pre-pregnancy BMI on GWG, we encourage further investigation of preconception lifestyle interventions to support individuals meeting a healthy BMI before pregnancy.

## Figures and Tables

**Table 1:** Demographic characteristics of 2914 women in the Pregnancy Environment and Lifestyle Study (PETALS) who delivered between 2014 and 2019.

Characteristic	*n* = 2914
Age at delivery, years	
18–24	444 (15.2%)
25–29	754 (25.9%)
30–34	1072 (36.8%)
35 and older	644 (22.1%)
Nulliparous, *n* (%)	1339 (46%)
Pre-pregnancy BMI, kg/m^2^	27.0 (6.0)
Race and ethnicity, *n* (%)	
Asian/Pacific Islander	690 (24%)
Black	280 (9.6%)
Hispanic	1175 (40%)
Other/Unknown	94 (3.2%)
White	675 (23%)
Education level, *n* (%)	
Less than high school	79 (2.7%)
High school graduate or GED	324 (11%)
Some college	1069 (37%)
Completed 4-year college or higher	1439 (49%)
Household income, *n* (%)	
Less than USD 50,000 per year	910 (32%)
USD 50,000 to USD 99,999 per year	911 (32%)
USD 100,000 to USD 149,999 per year	531 (18%)
USD 150,000 and greater per year	520 (18%)
Total energy intake, kcal/day	1567.7 (734.8)
Physical activity, METs/week	10.9 (14.2)
Dietary quality index score, mean (SD)	
HEI-2010	71 (10)
DASH	24.0 (4.4)
aMED	4.01 (1.79)
EDIP	−0.05 (0.45)
Gestational weight gain rate, *n* (%)	
Inadequate	414 (14%)
Met	860 (30%)
Excessive	1640 (56%)

**Table 2:** Adjusted * relative risk of excessive and inadequate gestational weight gain rate by diet quality defined by HEI-2010, DASH, aMED, and EDIP.

	Diet Quality Index			
	HEI-2010 ^1^	DASH ^2^	aMED ^3^	EDIP ^4^
**Excessive GWG**				
Good quality	1 (reference)	1 (reference)	1 (reference)	1 (reference)
Poor quality	**1.03 (1.00, 1.06)**	1.02 (0.99, 1.05)	1.01 (0.98, 1.04)	1.00 (0.97, 1.03)
**Inadequate GWG**				
Good quality	1 (reference)	1 (reference)	1 (reference)	1 (reference)
Poor quality	1.00 (0.92, 1.09)	0.94 (0.86, 1.03)	1.04 (0.94, 1.15)	0.98 (0.89, 1.08)

Bolded estimates denote *p* ≤ 0.05. * All models adjusted for physical activity, education level, parity, household income, race-ethnicity, age at delivery, pre-pregnancy BMI, and total energy intake. ^1^ Maximum score is 100. Higher score indicates better alignment with dietary recommendations. Good-quality diet was defined by the 75th percentile or higher (≥78.7). ^2^ Maximum score is 40. Higher score indicates better alignment with dietary recommendations. Good-quality diet was defined by the 75th percentile or higher (≥28). ^3^ Maximum score is 8. Higher score indicates better alignment with dietary recommendations. Good-quality diet was defined by the 75th percentile or higher (≥6). ^4^ No maximum score. Lower score indicates less inflammatory diet. Good-quality diet was defined by the 25th percentile or lower (≤−0.20).

**Table 3:** Adjusted * relative risk of excessive gestational weight gain rate by diet quality defined by HEI-2010, stratified by pre-pregnancy BMI.

	Pre-Pregnancy BMI		
	BMI < 25.0 *n* = 1066	25.0 ≤ BMI ≤ 29.9 *n* = 763	BMI ≥ 30.0 *n* = 671
**Excessive GWG**			
Good quality	Ref	Ref	Ref
Poor quality	**1.05 (1.00, 1.10)**	1.04 (0.97, 1.06)	1.01 (0.97, 1.06)

Bolded estimates denote *p* ≤ 0.05. * All models adjusted for physical activity, education level, parity, household income, race-ethnicity, age at delivery, and total energy intake.

**Table 4:** Adjusted * relative risk of excessive gestational weight gain rate by diet quality defined by HEI-2010, stratified by race and ethnicity.

	Race and Ethnicity				
	Asian and Pacific Islander *n* = 567	Black, *n* = 246	Hispanic, *n* = 1001	Other or Unknown, *n* = 86	White, *n* = 600
**Excessive GWG**					
Good quality	Ref	Ref	Ref	Ref	Ref
Poor quality	1.00 (0.94, 1.06)	**1.14 (1.02, 1.28)**	0.99 (0.95, 1.03)	1.15 (0.99, 1.35)	**1.07 (1.01, 1.12)**

Bolded estimates denote *p* ≤ 0.05. * All models adjusted for physical activity, education level, parity, household income, race-ethnicity, age at delivery, pre-pregnancy BMI, and total energy intake.

## Data Availability

Data described in the manuscript will not be made available due to their containing information that could compromise the privacy of research participants. The analytic code is available upon reasonable request from the corresponding author, E.F.L.

## Supplementary Materials

The following supporting information can be downloaded at: https://www.mdpi.com/article/10.3390/nu15040835/s1, Table S1: Adjusted relative risk of excessive gestational weight gain rate by diet quality quartiles defined by HEI-2010, DASH, aMED, and EDIP; Table S2: Adjusted relative risk of inadequate gestational weight gain rate by diet quality quartiles defined by HEI-2010, DASH, aMED, and EDIP.

## Abbreviations

aMED	Alternate Mediterranean Diet
DASH	Dietary Approaches to Stop Hypertension
EDIP	Empirical Dietary Inflammatory Pattern
EHR	Electronic health records
GWG	Gestational weight gain
HEI	Healthy Eating Index
IOM	Institute of Medicine
