# Supplementary material for: Dietary Quality Indices in Early Pregnancy and Rate of Gestational Weight Gain among a Prospective Multi-Racial and Ethnic Cohort
Source: Nutrients. 2023 Feb 6;15(4):835. doi: 10.3390/nu15040835 (PMC9961419; doi:10.3390/nu15040835)
Supplement: Supplementary file 1 [file nutrients-15-00835-s001.zip › nutrients-2119081-supplementary.pdf]

nutrients

MDPI

Supplementary Materials

Dietary Quality Indices in Early Pregnancy and Rate of Gestational Weight Gain among a Prospective Multi-Racial and Ethnic Cohort

Emily F. Liu *, Yeyi Zhu, Assiamira Ferrara and Monique M. Hedderson

Table S1. Adjusted* relative risk of excessive gestational weight gain rate by diet quality quartiles defined by HEI-2010, DASH, aMED, and EDIP.

	Dietary Pattern			
	**HEI-2010** [1]	**DASH** [2]	**aMED** [3]	**EDIP** [4]
**Quartile 1**	(37 to 65)<br>**1.04 (1.00, 1.07)**	(12 to 21)<br>1.00 (0.97, 1.04)	(0 to 3)<br>1.00 (0.96, 1.04)	(−5.95 to −0.21)<br>*Ref*
**Quartile 2**	(65 to 72)<br>**1.05 (1.01, 1.08)**	(21 to 24)<br>1.02 (0.99, 1.06)	(3 to 4)<br>1.00 (0.96, 1.04)	(−0.21 to −0.01)<br>0.99 (0.96, 1.03)
**Quartile 3**	(72 to 79)<br>1.01 (0.98, 1.05)	(24 to 27)<br>1.03 (0.99, 1.06)	(4 to 5)<br>1.02 (0.99, 1.05)	(−0.01 to 0.17)<br>0.98 (0.95, 1.01)
**Quartile 4**	(79 to 96)<br>*Ref*	(27 to 38)<br>*Ref*	(5 to 8)<br>*Ref*	(0.17 to 3.17)<br>1.00 (0.97, 1.03)

Bolded estimates denote a p-value ≤ 0.05.

*All models adjusted for physical activity, education level, parity, household income, race-ethnicity, age at delivery, pre-pregnancy BMI, and total energy intake. .

[1] Maximum score is 100. Higher score indicates better alignment with dietary recommendations.

[2] Maximum score is 40. Higher score indicates better alignment with dietary recommendations.

[3] Maximum score is 8. Higher score indicates better alignment with dietary recommendations.

[4] No maximum score. Lower score indicates less inflammatory diet.

Table S2. Adjusted* relative risk of inadequate gestational weight gain rate by diet quality quartiles defined by HEI-2010, DASH, aMED, and EDIP.

	Dietary Pattern			
	**HEI-2010** [1]	**DASH** [2]	**aMED** [3]	**EDIP** [4]
**Quartile 1**	(37 to 65)<br>0.92 (0.82, 1.03)	(12 to 21)<br>0.96 (0.85, 1.08)	(0 to 3)<br>1.07 (0.94, 1.21)	(−5.95 to −0.21)<br>*Ref*
**Quartile 2**	(65 to 72)<br>0.98 (0.88, 1.10)	(21 to 24)<br>0.97 (0.87, 1.09)	(3 to 4)<br>1.05 (0.92, 1.19)	(−0.21 to -0.01)<br>0.92 (0.83, 1.03)
**Quartile 3**	(72 to 79)<br>0.96 (0.86, 1.07)	(24 to 27)<br>0.91 (0.82, 1.02)	(4 to 5)<br>1.04 (0.94, 1.15)	(−0.01 to 0.17)<br>0.90 (0.81, 1.00)
**Quartile 4**	(79 to 96)<br>*Ref*	(27 to 38)<br>*Ref*	(5 to 8)<br>*Ref*	(0.17 to 3.17)<br>0.93 (0.84, 1.03)

Bolded estimates denote a p-value ≤ 0.05.

*All models adjusted for physical activity, education level, parity, household income, race-ethnicity, age at delivery, pre-pregnancy BMI, and total energy intake. .

[1] Maximum score is 100. Higher score indicates better alignment with dietary recommendations.

[2] Maximum score is 40. Higher score indicates better alignment with dietary recommendations.

[3] Maximum score is 8. Higher score indicates better alignment with dietary recommendations.

[4] No maximum score. Lower score indicates less inflammatory diet.
